# Evaluation of Traditional Medicines for Neurodegenerative Diseases Using *Drosophila* Models

**DOI:** 10.1155/2014/967462

**Published:** 2014-03-26

**Authors:** Soojin Lee, Se Min Bang, Joon Woo Lee, Kyoung Sang Cho

**Affiliations:** Department of Biological Sciences, Konkuk University, Seoul 143-701, Republic of Korea

## Abstract

*Drosophila* is one of the oldest and most powerful genetic models and has led to novel insights into a variety of biological processes. Recently, *Drosophila* has emerged as a model system to study human diseases, including several important neurodegenerative diseases. Because of the genomic similarity between *Drosophila* and humans, *Drosophila* neurodegenerative disease models exhibit a variety of human-disease-like phenotypes, facilitating fast and cost-effective *in vivo* genetic modifier screening and drug evaluation. Using these models, many disease-associated genetic factors have been identified, leading to the identification of compelling drug candidates. Recently, the safety and efficacy of traditional medicines for human diseases have been evaluated in various animal disease models. Despite the advantages of the *Drosophila* model, its usage in the evaluation of traditional medicines is only nascent. Here, we introduce the *Drosophila* model for neurodegenerative diseases and some examples demonstrating the successful application of *Drosophila* models in the evaluation of traditional medicines.

## 1. Introduction

The fruit fly* Drosophila* is considered the most useful animal genetic model, because of its high fecundity, short life cycle, and low cost of maintenance.* Drosophila* eggs grow into fertile adults within two weeks, and the females lay about 800 eggs in their lifetime [1]. Moreover, because they are tiny, they can be maintained in a small space, thereby lowering research costs. In addition, fruit flies can be quickly modified and tested, which offers several advantages over the more frequently used cell culture systems or mouse models that are expensive and require more time for experimental manipulation.

In addition, work with* Drosophila* mutants benefits from a legacy of powerful genetic tools for investigating genes of interest, screening for interacting proteins, and establishing tissue-specific and temporally regulated expression of foreign genes. An important method used in genetic studies is* P*-element-mediated mutagenesis that allows the creation of genome-wide mutations. Specifically, the* P*-element is a* Drosophila* transposon that has the ability to excise itself from and insert itself into various locations within the genome [2], thereby disrupting specific genes based on the presence of insertion sites. Currently, a large majority of the* Drosophila* genes, including most of the orthologs of neurodegenerative disease-causing genes, has been associated with at least one* P*-element mutant through several genome-wide* P*-element insertion projects [3, 4].

In contrast to* P*-element-mediated gene disruption, the yeast-based* UAS-GAL4* system is a method for activating gene expression [5]. The expression of the gene of interest is linked to an* upstream activation sequence* (*UAS*) modulated by the GAL4 protein, which is regulated by a tissue-specific promoter. In this system, the* UAS* is an enhancer that is specifically targeted by the GAL4 protein; however,* Drosophila* lacks endogenous targets for the* UAS*. The* UAS-GAL4* system is an efficient bipartite approach in the activation of gene expression [6]. One of the advantages of this system is that disease-causing toxic genes, such as* A*β*42* and mutant**α*-synuclein* (**α*-syn*), are only expressed when the GAL4 protein is bound to the* UAS* located upstream from the genes. This allows flies carrying the inactivated form of a toxic gene to survive normally. Another advantage of this system is that the effects of various genes can be studied through their overexpression or aberrant expression in various tissues and/or developmental stages using an array of known tissue-specific promoters. The analysis of the* Drosophila melanogaster* genome revealed the existence of orthologs for about 75% of human disease genes [7]. This striking genetic similarity has extended the use of* Drosophila* from basic developmental studies to effective modeling of human diseases. Moreover,* Drosophila* models can also be used for rapid screening of dietary components, drugs, and drug-administration regimens. The current review focuses on the utility of* Drosophila* neurodegenerative disease models for studying Chinese traditional medicines and the potential advantages of these models.

## 2. *Drosophila* as a Model System for Neurodegenerative Diseases

### 2.1. Representative* Drosophila* Models of Alzheimer's Disease

*Drosophila* has emerged as an excellent model system for a variety of neurodegenerative diseases including Alzheimer's disease (AD) and Parkinson's disease (PD) because of genetic homology, ease of genetic manipulation, and well-conserved disease-associated genes.* Drosophila* geneticists have successfully used these models to identify many novel disease-associated genes, which sheds light on our understanding of the pathology of these diseases.

**Figure 1:**
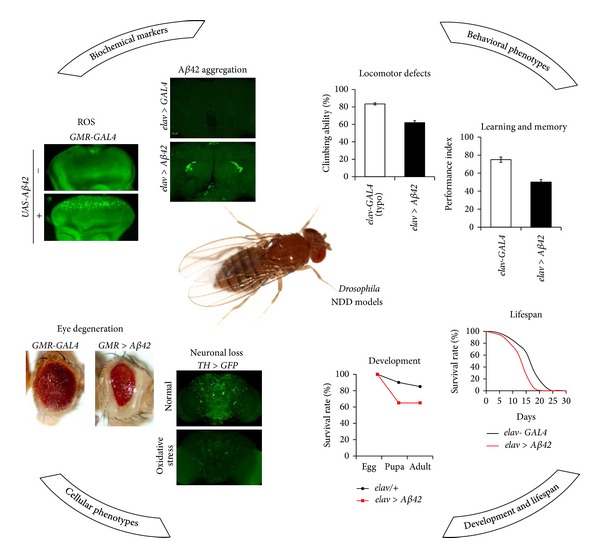
Representative neurological phenotypes of the* Drosophila* neurodegenerative disease models.* elav-GAL4*,* GMR-GAL4*, and* TH-GAL4* are drivers that regulate gene expression in neurons, developing eyes, and DA neurons, respectively.* elav* >* A*β*42* and* GMR *>* A*β*42* represent flies expressing* A*β*42* in the neurons and developing eyes, respectively.* TH* >* GFP* represents flies expressing GFP in the DA neurons. GFP: green fluorescent protein; GMR: glass multimer reporter; NDD: neurodegenerative disease; TH: tyrosine hydroxylase; UAS: upstream activation sequence.

AD is the most common neurodegenerative disease, which causes a deficiency in memory and other cognitive functions [8]. The primary event in AD pathogenesis is the accumulation of amyloid *β*-peptide 42 (A*β*42), a form of *β*-amyloid precursor protein (APP) proteolytically processed by *γ*-secretase [8]. Aggregates of abnormally phosphorylated tau, a microtubule-binding protein, are also shown to be closely associated with neuronal loss in AD [9]. The* Drosophila* genome contains genes that encode orthologs of APP, tau, and four major protein components of *γ*-secretase (presenilin, nicastrin, APH-1, and PEN-2). Transgenic flies expressing human* A*β*42* or* tau *ectopically developed late-onset neuronal degeneration and had a shortened lifespan [10, 11].* Drosophila* AD models present various easily visible and quantifiable phenotypes such as eye degeneration, developmental defects, shortened lifespan, locomotor defects, increased oxidative stress sensitivity, and learning and memory defects, which make it suitable for* in vivo* genetic screening (Figure 1). The experimental methods for analyzing these phenotypes are described in the following section.

Based on genetic screening using* Drosophila* models of AD, several biochemical processes such as secretion, cholesterol homeostasis, and regulation of chromatin structure have been found to be involved in mediating the toxic effects of A*β*42 [12, 13]. In* tau*-expressing models, kinases and phosphatases comprised the major classes of modifiers of the tauopathy [14], and cytoskeleton proteins and molecular chaperones have been identified as modulators of mutant tau-induced neurodegeneration [15]. More recently, DNA damage-activated checkpoint kinase 2, histone deacetylase 6, and epidermal growth factor receptor (EGFR) have been reported to be implicated in AD pathologies in* Drosophila* AD models [16–18].

Moreover, several* in vivo* reporter systems for measuring APP *γ*-secretase activity were developed in* Drosophila*. Among them, the transgenic system consisting of the human APP and the yeast GAL4 fusion protein under the expression of the eye-specific* glass multimer reporter* (*GMR*) promoter has been applied as a powerful genetic screening tool for isolating *γ*-secretase activity-regulating molecules [19]. In this reporter system, in the presence of *γ*-secretase activity, the intracellular domains of APP and GAL4 translocate to the nucleus and induce* GRIM* expression, which results in cell death in the eye. Therefore, genetic or pharmacological modulators of *γ*-secretase activity can be screened by simply observing the eye degeneration phenotype. Several genetic modulators of *γ*-secretase activity were also identified using this reporter system [20–22].

### 2.2. Representative* Drosophila* Models of Parkinson's Disease

PD is the second most common neurodegenerative disease, the representative clinical feature being motor dysfunction caused by the loss of dopaminergic (DA) neurons in the substantia nigra [23]. The pathological hallmark of idiopathic PD is the formation of Lewy bodies, in which *α*-syn protein accumulates abnormally [24]. Although most PD patients have a sporadic disease, recent studies identified several familial PD-related genes including* SNCA* (encoding *α*-syn protein),* UCH-L1 *(encoding ubiquitin C-terminal hydrolase-like 1 protein),* PRKN* (encoding parkin protein),* LRRK 2 *(encoding leucine-rich repeat kinase 2 protein),* PINK 1 *(encoding PTEN-induced kinase protein), and* DJ-1 *(encoding DJ-1 protein) [25]. The* Drosophila* genome contains homologs of all the PD-linked genes except for* SNCA*. However, expression of human *α*-syn in fly neurons formed Lewy body-like filamentous intraneuronal inclusions, and the mutant flies showed loss of DA neurons and a locomotor defect phenotype [26]. Subsequently, several mutants of* parkin*,* DJ-1*,* Pink1*, and* LRRK2* have been generated in* Drosophila* and their phenotypes characterized [25]. These* Drosophila* models of PD show various PD-like neurological phenotypes such as locomotor defects, sensitivity to oxidative stress, developmental defects, and reduced lifespan (Figure 1) [27–35]. Moreover, the critical role of mitochondria in the pathogenesis of PD has been discovered using these models [36]. For example, parkin and PINK1 play an important role in mitochondrial function and genetically interact in this pathway [32, 33, 37, 38], and DJ-1 is also involved in mitochondrial function [36, 39, 40].

These models have been adopted to discover the molecular mechanisms underlying PD pathogenesis [41–44]. For example, screening for genetic modifiers of* Pink1*/*parkin* identified several factors that function in oxidative stress, innate immune response, polyubiquitination, signal transduction, and N-glycosylation [41, 43]. More recently, screening of chromosomal deletions combined with a genome-wide RNAi screen identified TRAP1, a mitochondrial chaperone protein, as a suppressor of a disease-linked form of *α*-syn ([A53T] *α*-syn)-induced DA neuron loss and behavioral defect [44]. In this study, the inhibitory effect of TRAP1 on *α*-syn toxicity was also confirmed in several mammalian cell types including rat primary cortical neurons, suggesting that the role of TRAP1 in the health of DA neurons is well conserved in insects [44]. Moreover, subsequent studies showed that TRAP1 rescues the mitochondrial impairments of both* parkin* and* PINK1* mutants [45, 46].

### 2.3. Representative Neurological Phenotypes of* Drosophila* Models of AD and PD

*Drosophila *neurodegenerative disease models show a variety of phenotypes, which are very similar to the symptoms of human patients and closely linked with the neuropathology of the diseases. These phenotypes include a wide range of biological processes, from cellular phenotypes to behaviors (Figure 1). These prominent and easily observable phenotypes make* Drosophila* a valuable model for drug screening and discovery of novel disease-associated genes.

#### 2.3.1. Accumulation of A*β*42 and *α*-syn

One of the major characteristics of AD is the accumulation of amyloid protein in the cerebral cortex [8]. Mutation in* APP*,* Presenilin 1*, and* Presenilin 2* genes or other factors increases A*β*42 production and accumulation [8]. Consequently, increased A*β*42 oligomerization and deposition injure the neurons, causing neuronal dysfunction and cell death. These AD-like phenotypes can also be observed in* Drosophila* AD models. Overexpression of* A*β*42* in the nervous system induces neuronal loss accompanied by the accumulation of A*β*42 in the adult brain. Similarly, PD as a neurodegenerative disease is characterized by the loss of nigrostriatal DA neurons and the accumulation of Lewy bodies in neurons [47]. Overexpression of **α*-syn* gene in the nervous system of the fly model results in the death of DA neurons and the formation of Lewy body-like filamentous intraneuronal inclusions [26].

#### 2.3.2. Increased Reactive Oxygen Species Level

Excessive reactive oxygen species (ROS) induce tissue damage and cell death by oxidizing lipids, proteins, and DNA [48]. The brain is particularly sensitive to oxidative stress and it has been reported that oxidative stress is increased in the brain in the presence of neurodegenerative diseases [49]. The increased ROS damage has been identified in specific brain areas such as the cortex and hippocampus of AD patients and in the substantia nigra of PD patients [49]. As in human patients, the ROS level in the brain increased in* Drosophila* AD and PD models [31, 50, 51]. Moreover, flies expressing* A*β*42 *or* tau* ^*R406W*^ are more sensitive to oxidative stress, and genetic and pharmacological upregulation of antioxidant defenses suppressed the neurological impairments in the* A*β*42*- or* tau* ^*R406W*^-expressing flies [13, 52].

The level of oxidative stress was measured in* Drosophila* neurodegenerative disease models by several methods [31, 50, 53]. Among them, dihydroethidium (DHE) staining is one of the simplest, in which the DHE penetrates the cell membrane and forms 2-hydroxyethidium by interacting with intracellular O_2_ [54]. Because the 2-hydroxyethidium is fluorescent at specific wavelengths, increased expression of this fluorescence indicates rising levels of oxidative stress [54].

#### 2.3.3. Eye Degeneration

Although the central nervous system is the main target of neurodegenerative diseases such as AD, PD, and Huntington's disease (HD), functional defects in these diseases are not restricted to the brain [55, 56]. For example, extensive ganglion cell loss was observed in the central retina of AD patients [57], and visual dysfunction caused by retinal degeneration has been found in multiple transgenic AD mouse lines [55]. Thus, tissues other than that of the brain can be used to identify the function of genes related to neurodegenerative diseases.

Eyes are prominent organs in the body of* Drosophila*. Therefore, an ocular phenotype is easily distinguishable and facilitates simple, easy, and efficient genetic or pharmacological screening. Moreover, the developing* Drosophila* eye contains the photoreceptor neurons.* Drosophila* has two compound eyes, each consisting of about 800 ommatidia and bristles. These ommatidia are arranged very regularly [58]. Using the* UAS-GAL4 *system, the expression of a human disease-related transgene in the fly eye creates a fly model for neurodegenerative disease as well as helps to discover the function of the gene. For example, overexpression of the* A*β*42* and* tau* genes involved in AD or the **α*-syn* gene involved in PD induces apoptotic eye degeneration, reduced eye size, and deformed ommatidia [26, 59].

#### 2.3.4. Neuronal Loss

One of the most prominent neuronal degeneration phenomena is the loss of DA neurons in PD. Although the numbers of DA neurons are relatively few, these neurons play a crucial role in motor control, motivation, and working memory in the substantia nigra pars compacta of the midbrain by synthesizing dopamine [60]. Surprisingly, DA neurons and dopamine-associated pathways are well conserved between humans and* Drosophila* [61–63]. In addition, the DA neurons appeared to be destroyed in many of the mutant fly models of PD [26, 28, 34, 37, 40, 42].

On the other hand, one of the major pathomorphological changes of AD is neuronal degeneration in the frontal and temporal lobes and in the hippocampus, the latter being a crucial region for learning and memory [64, 65]. Interestingly, the mushroom body performs a crucial function in learning and memory in* Drosophila* [66, 67], similar to the hippocampus in humans. Severe neuronal loss occurs in the Kenyon cell layer, intrinsic neurons composing the mushroom body, in the AD model fly [11]. This suggests that* Drosophila* is an appropriate model for recapitulating neuronal loss in humans. Therefore, the loss of DA and hippocampal neurons, which are important disease phenotypes, can be explored by using fly models, leading to insights into the pathogenesis and mechanisms underlying PD and AD.

#### 2.3.5. Developmental Defects and Shortened Lifespan

Human neurodegenerative diseases are mostly late-adult onset disorders in which proteins such as *α*-syn and A*β*42 aggregate with age, resulting in toxicity [68, 69]. However,* Drosophila* neurodegenerative disease models artificially created for experiments are thought to have toxic protein aggregation beginning in the embryonic stage, not the adult stage. This results in developmental defects in the flies. For example,* A*β*42* expression in neurons induces apoptosis, thereby reducing the survival rate [70]. Despite these limitations, dramatic phenotypes from various stages, from larva to early adult, can be produced and are useful for determining the toxic activity of disease-causing proteins such as *α*-syn and A*β*42 [71, 72].

Aging is a comprehensive phenomenon that results from constant physiological degeneration over the lifetime of almost all organisms [73, 74]. Because of this, neurodegenerative diseases carry a risk of reduction in lifespan [74]. In accordance with this expectation, many studies have reported that the lifespan of patients with neurodegenerative diseases such as AD, PD, and HD is decreased [75]. For example, people with dementia are two to four times more likely to die at a given age than individuals without dementia of the same age [76]. Interestingly, fly models of neurodegenerative disease also have a reduced lifespan compared to control flies [13, 77, 78]. This result can be interpreted as a parallel to those seen in human neurodegenerative disease patients.

Overall, the developmental defects and shortened lifespans of fly neurodegenerative disease models can also be used as representative phenotypes.

#### 2.3.6. Locomotor Defects

Most neurodegenerative diseases are generally characterized by an age-dependent loss of locomotor ability. PD especially is a movement disorder, four fundamental features of which are tremor at rest, rigidity, akinesia (or bradykinesia), and postural instability [23]. Although clinical criteria for the diagnosis of AD include insidious onset and progressive impairment of memory and other cognitive functions [79], disorders of movement such as rigidity, slowness, and impaired gait have commonly been observed to accompany AD [80]. Consistently, most neurodegenerative disease fly models show reduced exercise ability, as observed in human patients [11, 26, 27, 70, 81, 82]. Climbing assay is an experiment to test movement ability based on a* Drosophila* trait, namely, the natural tendency to go against gravity [26, 83]. In this method, exercise ability is determined as the ratio of the number of flies that move to the top of their container to the total number of flies. Using this method, our laboratory and others have established that neurodegenerative disease model flies exhibit a locomotor defect and that the defect becomes more pronounced with age [15, 26, 84]. Therefore, the locomotor defect of* Drosophila* model flies can also be used as one of the representative phenotypes.

#### 2.3.7. Learning and Memory

Defects of learning and loss of memory are the most devastating symptoms of AD [64]. In AD patients, brain regions involved in learning and memory formation, such as the frontal lobe, temporal lobe, and hippocampus, exhibit reduced size as the result of loss of neurons and degeneration of synapses [65]. This neuronal loss and synaptic degeneration are caused by the presence of plaques and tangles [85]. Consistently, several studies showed that* Drosophila* AD models are also impaired in learning and memory [11, 86–88], which makes* Drosophila* a useful model of the defective brain functions of AD.

Memory function and learning ability in* Drosophila* can be assessed by its olfactory sense or courtship behavior [89, 90]. The test using olfaction is routinely performed in a T-maze apparatus [71]. In this tool, two different odors are used. Flies receive an electric shock in the presence of the first odor but not in the presence of the second odor. To test the learned memory of the odor, flies are moved to the T-maze choice point, between the sources of the two odors. After training, about 95% of the flies avoided the electric shock-associated odor. Using this method, learning and memory ability can be tested based on whether the fly associates the odor with the shock and avoids it or not. Several studies have thereby shown that* Drosophila* AD models are impaired in learning and memory [11, 86–88].

### 2.4. Use of* Drosophila* Models for Drug Discovery

The traditional drug discovery process is based on the “one disease-one target” hypothesis, in which high-throughput drug screening approaches are mostly based on* in vitro* screening platforms [71]. However, these cannot reflect the* in vivo* situation that needs to consider absorption, distribution, metabolism, excretion, and toxicity [71]. Therefore, the use of a simple* in vivo* model like* Drosophila* as a whole-animal primary screening platform would greatly increase the success rate in the drug discovery process.

Several advantages associated with the use of* Drosophila* make it a suitable model organism for drug screening. For instance,* Drosophila* is smaller than other model organisms such as mice and rats, requiring less space and a lower budget. Therefore, it is possible to breed a large number of flies in relatively smaller spaces, enabling the experimental figures to be statistically more significant. The other beneficial feature is the shorter time required to perform experiments with* Drosophila*. The lifespan of mice is more than 24 months, whereas that of* Drosophila* is less than 3 months under laboratory conditions. Moreover, the time needed to express the disease-like phenotypes in* Drosophila* models is much shorter than that in mouse models, which makes it possible to measure drug efficacy in a reasonable period. Based on these advantages,* Drosophila* neurodegenerative disease models have been extensively adopted to screen or validate drugs.

Because accumulation of A*β*42 in the brain is an important cause of AD [8], blocking this accumulation has been considered a promising way to treat AD. To date, several molecules including curcumin, 1,4-naphthoquinone-2-yl-L-tryptophan, glutaminyl cyclase inhibitor, and D737 have been reported to block the accumulation to some extent, thereby reducing the AD-like phenotypes in* Drosophila* AD models [91–95]. D737 in particular was identified by high-throughput screening for inhibitors of A*β*42 aggregation using a collection of 65,000 small molecules in cell culture. Its efficacy was subsequently evaluated with a* Drosophila* AD model [95].

Another factor that influences the formation of A*β*42 oligomers is the abnormal expression of *γ*-secretase, which has been associated with the increased level of A*β*42 and the pathology of AD [8]. Therefore, *γ*-secretase inhibitors are considered candidate therapeutic agents for AD. As expected, a study has reported that treatment with a *γ*-secretase inhibitor, L-685,458, significantly reduced AD-like phenotypes such as memory dysfunction, defects of motor ability, and neuronal cell death in a* Drosophila* AD model that expresses both human* APP* and the **β*-secretase* (*BACE*) gene [96].

In addition, AD patients show abnormal signal transductions such as activation of c-Jun N-terminal kinase (JNK), extracellular signal-regulated kinase (ERK), and glycogen synthase kinase-3, which induce neurological impairments including cell death and memory defects [97–100]. Therefore, inhibitors of these signaling molecules are potentially therapeutic drugs. Indeed, when* Drosophila *AD models were treated with inhibitors of JNK or ERK, their AD-like phenotypes were alleviated [70, 101].

The efficacies of a variety of potential PD drugs also have been tested in* Drosophila* PD models. Among them, several drugs including mannitol, cinnamon extract precipitate, isorhynchophylline, and inhibitors of the silent information regulator 2 (Sir2) were shown to inhibit *α*-syn aggregation [102–105]. Mannitol, a 6-carbon polyol isolated from* Fraxinus ornus* [106], possesses blood-brain barrier-disrupting properties, and treatments with mannitol reduced motor defects in* Drosophila* PD models [105]. A herbal substance, cinnamon extract precipitate, was also reported to ameliorate PD model phenotypes and to significantly decrease the accumulation of agglomerated *α*-syn in the brain [103]. Isorhynchophylline, which is a natural alkaloid isolated from the Chinese herbal medicine* Uncaria rhynchophylla*, promoted the degradation of *α*-syn in neuronal cells by inducing autophagy [104]. In addition, inhibitors of Sir2 suppressed *α*-syn toxicity and aggregation forms in a* Drosophila* PD model [102].

Neurodegenerative diseases including PD are correlated with oxidative stress, and ROS have been reported to cause neuronal injury [107]. Therefore, drugs that possess antioxidant properties may have beneficial effects against neurodegenerative diseases. Indeed, L-ascorbic acid (vitamin C) has antioxidant properties and partially rescues the PD-like phenotypes of the* Drosophila* PD model [108]. Another group of promising antioxidant drugs includes the polyphenols. The survival and motor defects of PD model flies were rescued by polyphenol treatments [109], which suggests that antioxidant therapy is a promising way to treat neurodegenerative disease including PD.

## 3. Effects of Traditional Medicine on* Drosophila* Models of Neurodegenerative Diseases

Although* Drosophila* is one of the most well studied model animals and has been extensively used to create neurodegenerative disease models, a surprisingly small number of studies investigating the beneficial effects of traditional medicine in* Drosophila* models of neurodegenerative diseases has been performed to date. This might be due to the disparity in metabolic and physiological systems between insects and mammals, which makes it challenging to study the effect of herbal medicine in* Drosophila*. However, several recent studies described in this review have demonstrated the potential of* Drosophila* as a useful model for testing the effects of traditional medicines on neurodegenerative diseases.

### 3.1. Effects of SuHeXiang Wan on* Drosophila* AD Models Expressing Human A*β*42

SuHeXiang Wan (SHXW) is a Chinese traditional medicinal prescription that has been used for treating depression, seizures, infantile convulsion, and stroke [110]. The original prescription of SHXW consists of 15 crude herbs [110]. Among them, nine herbs have the term “Xiang” (fragrance) in their Chinese names, which implies that the essential oils in SHXW may be important for exerting its beneficial effects. Recently, a modified version of SHXW is being used because some constituents of the original prescription, such as* cinnabar*,* Styrax benzoin*,* Saussurea lappa*, and* Boswellia carterii*, have been prohibited due to their toxicity [84, 111]. A previous study showed that oral administration of SHXW reduced stress-hormone levels in an immobilization-stress assay using rodents [112]. In a later study, inhalation of essential oils from SHXW was found to inhibit convulsions by acting on GABAergic neurotransmission, GABA transaminase activity, and brain lipid peroxidation.

**Figure 2:**
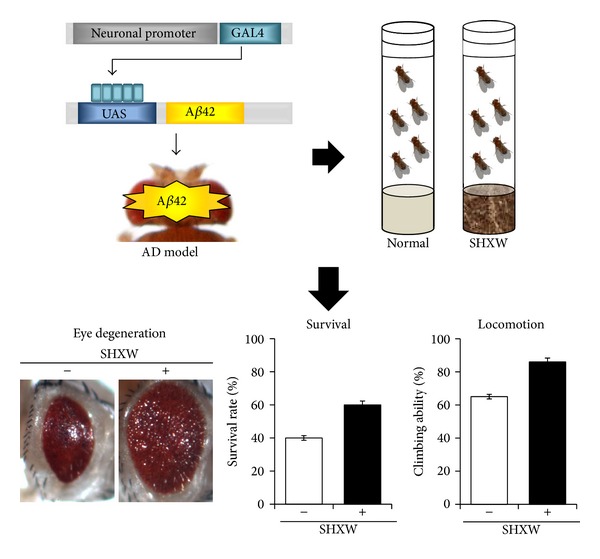
The effects of SHXW on the neurological phenotypes of a* Drosophila* AD model. AD: Alzheimer's disease; SHXW, SuHeXiang Wang (adapted from [101]).

The beneficial effects of a modified version of SHXW, KSOP1009, on AD have been investigated in* Drosophila* models [84, 101] (Figure 2). In these studies, SHXW was extracted with* n*-hexane, and the extract was added to the standard cornmeal-soybean fly medium. Feeding with SHXW extract strongly suppressed the eye-degeneration phenotype induced by human* A*β*42* expression in the flies [84].* A*β*42*-induced cell death in the developing eye imaginal disc was also inhibited by SHXW intake [84]. This is possibly due to the suppression of* A*β*42*-mediated neurotoxic effects as observed in mammalian cells. However, in some ways these results from the* Drosophila* eye model are more relevant to human disease than the results from mammalian cells, because these results strongly suggest that SHXW enters the animal body from the gut, targets neurons, and exerts its protective effect not only on cells but also on tissues. Accordingly, SHXW intake significantly improved the developmental defects and motor activity of flies expressing* A*β*42 *in neurons [84]. The neuroprotective effect of SHXW against A*β*42 insult observed in* Drosophila* can be replicated in mammalian cells and mice. In the cell studies, the viability of A*β*42-treated SH-SY5Y cells in the SHXW essential oil-treated group was much higher than that of the group receiving only A*β*42 [111, 113]. Moreover, both inhalation and oral administration of SHXW essential oil alleviated A*β*42-induced memory impairment in mouse AD models [111, 113]. These results indicate the usefulness of the* Drosophila* model for screening or testing traditional remedies for neurodegenerative diseases.

In addition to their use in testing the efficacy of SHXW,* Drosophila* models were also used to study the molecular mechanisms by which the medicine exerts its beneficial effects. It has been well established that mitogen-activated protein kinases (MAPKs), JNK, ERK, and p38MAPK are hyperactivated in the brains of animal models of AD and patients who chronically express* A*β*42* [98, 114]. Consistent with the human patient and mammalian models, chronic expression of* A*β*42* in* Drosophila* resulted in the hyperactivation of JNK and ERK [70, 84, 101, 115]. Moreover, inhibition of the JNK or EGFR/ERK signaling pathways ameliorated the A*β*42-induced defective phenotypes, including the* A*β*42*-induced lethality and locomotor defects [70, 101]. These results suggest that not only the neurological phenotypes but also the pathophysiology of AD are well conserved in* Drosophila* AD models.

SHXW treatment suppressed the eye degeneration induced by activation of JNK, which is closely associated with the A*β*42-induced cytotoxicity [84]. Moreover, the level of JNK phosphorylation in eye imaginal discs overexpressing JNK kinase (JNKK) was decreased by SHXW treatment [84], which suggests that SHXW has inhibitory activity against JNKK. The inhibitory effect of SHXW on the JNK signaling pathway was confirmed in a study using the A*β*42-treated mouse model, in which the inhalation of SHXW essential oil completely suppressed the A*β*42-induced phosphorylation of JNK [111]. This suggests that the pathophysiology of fly AD models is similar to that of the mouse. In a series of studies, SHXW also exhibited therapeutic effects on the neurological phenotypes in* Drosophila* AD models by inhibiting the EGFR/ERK pathway [101]. SHXW intake significantly decreased ERK phosphorylation levels in the head and suppressed a wing vein formation defect in* A*β*42*-expressing flies [101]. These studies suggest that SHXW may contain some components that act as inhibitors of the JNK and EGFR/ERK signaling pathways. For example,* Myristica fragrans*, a component herb of SHXW, contains macelignan, which has been reported to inhibit cisplatin-induced hepatocytotoxicity by abolishing the phosphorylation of JNK and ERK [116]. Additionally, SHXW suppressed A*β*42-induced glial cell proliferation [117], which may further indicate its association with the pathophysiological neuroinflammation of the AD brain. Interestingly, glial cell proliferation induced by A*β*42 is not related to JNK or ERK activation [101], which suggests that SHXW has another mechanism besides JNK and ERK inhibition for providing neuroprotection against A*β*42-associated neuronal pathology. Based on the variety of neuronal phenotypes and pathophysiologies of* Drosophila* AD models, which are reasonably similar to those of human patients and the availability of numerous useful genetic tools in this organism, the various beneficial effects of SHXW were successfully evaluated. A series of studies showed that SHXW exerts its beneficial effects through various therapeutic pathways [84, 101, 111, 113]. This can be explained by the nature of SHXW, which is a mixture of several herbs like the other traditional Chinese medicines. As an herbal mixture medicine, SHXW may contain numerous beneficial components that are effective against AD. Therefore, SHXW may have a higher probability of treating a disease that has complex pathological pathways, such as AD. This idea is supported by the results from several recent studies, which showed that combination drug therapy is more effective than monotherapy for the treatment or prevention of AD [118–120]. This emphasizes the potential of traditional medicine as a combination drug therapy for neurodegenerative diseases.

### 3.2. Effect of* Gardenia jasminoides* Ellis Components and* Gastrodia elata* Blume Extract on* Drosophila* AD Models

*Gardenia jasminoides* Ellis is an evergreen shrub distributed widely in the tropical and subtropical regions of the world, growing on mountain slopes or roadsides as an ornamental plant [121].* Gardeniae fructus*, the dried ripe fruits of this plant, is widely used in Asian countries as a natural colorant and as a traditional Chinese medicine for its anti-inflammatory, analgesic, and antipyretic effects [122, 123].

It contains geniposide and crocin as its main components. These components have various beneficial effects including antioxidation and neuroprotection [124, 125]. Crocin antagonizes the inhibitory effect of ethanol on NMDA receptor-mediated long-term potentiation in rat hippocampal neurons [124] and inhibits the oxidative stress caused by serum/glucose deprivation in PC12 cells [125]. Moreover, crocetin, the aglycone of crocin, has been shown to have protective effects against retinal damage via inhibition of the increase in caspase-3 and -9 activities that occur after retinal damage [126].

Besides geniposide and crocin, various glucosides and quinic acid derivatives have been isolated from the fruits of* Gardenia jasminoides* Ellis [121, 127]. Their short-term-memory-enhancement activities were evaluated recently in an A*β* transgenic* Drosophila* model [121, 127]. Among 19 tested compounds, 13 showed short-term-memory-enhancement activities in AD flies [121, 127]. Interestingly, polyphenolics such as phenylpropanoid glycosides and lignans have been identified as neuroprotective agents in various neurodegenerative disease models including models of AD and PD, which suggests that Gardenia* jasminoides* Ellis may be beneficial in these diseases [128].

The neuroprotective effect of the aqueous extract of the rhizome of* Gastrodia elata* Blume (GE) on A*β*-induced toxicity was also investigated in* Drosophila* models [129]. Traditionally, the tubers of GE are widely used to treat some syndromes or diseases attributed to “wind blowing on the brain,” such as dizziness, convulsion, hypertension, and stroke, and the possible active ingredients are gastrodin, vanillin, and an extract of the fungus* Armillaria mellea* [130]. The beneficial effects of GE extract or its pure components on A*β*-induced toxicity have been demonstrated by* in vitro* studies [131, 132]. The ethyl ether fraction of GE protects against A*β* peptide-induced cell death in IMR-32 neuroblastoma cells [131], and the GE methanol extract, gastrodin, or 4-hydroxybenzyl alcohol (an aglycone of gastrodin) suppressed A*β*-induced cell death and showed a regulatory effect on endoplasmic reticulum stress proteins in BV-2 microglial-derived cells [132].

More recently, an* in vivo* study demonstrated a protective effect of GE aqueous extract against A*β*42-induced damage using* Drosophila* AD models [129]. In this study, both 1 and 5 mg of GE aqueous extract per gram of* Drosophila* medium significantly increased the median and maximum lifespan of A*β* flies by 12.0% and 26.9%, respectively, and improved the locomotor activity of A*β*42-expressing flies of various ages [129]. Moreover, the neurodegeneration in the ommatidia of eye-specific A*β*42-expressing flies was also reduced by GE aqueous extract treatment [129]. These results suggest that GE extract ameliorates the developmental and locomotor defects of A*β*42-expressing flies by protecting cells from A*β*42 cytotoxicity. Consistently, GE aqueous extract showed antiapoptotic and antioxidative effects against A*β*-induced damage in a dose dependent manner in mammalian PC12 cells [129].

### 3.3. Effect of Celastrol on a* Drosophila *PD Model

*Tripterygium wilfordii* Hook, also known as the Thunder God Vine, is a perennial vine that contains a variety of therapeutically active compounds such as terpenoids, alkaloids, and steroids [133, 134]. These compounds are traditional Chinese medicines, which have been used in the treatment of various diseases since the 1960s. Celastrol is a potent anti-inflammatory and antioxidant triterpene that is extracted from the root bark of* Tripterygium wilfordii*. Many studies have demonstrated that celastrol has anti-inflammatory and antioxidant effects in many* in vivo* models of diseases such as allergic asthma, amyotrophic lateral sclerosis, cancer, neurodegenerative diseases, multiple myeloma, and rheumatoid arthritis [135–141]. In particular, several studies have demonstrated that celastrol prevents the production of A*β*42 by reducing* BACE* expression through NF-*κ*B inactivation [138]. Celastrol also suppressed overactivation of microglia in the brain of a mouse model of AD, thereby significantly improving learning and memory [135, 138].

The powerful anti-inflammatory and antioxidant activities of celastrol also protected DA neurons and dopamine level in a* Drosophila* model of PD [137]. In this study, the effect of celastrol was evaluated* in vivo* by measuring the survival of DA neurons and the dopamine content in the brain of* DJ-1*α**RNAi model flies. Similar to patients with PD, the decreased DJ-1 level in DA neurons resulted in an age-dependent reduction in the number of DA neurons and in dopamine level [137]. RNAi based reduction in the expression of* DJ-1*α**in* Drosophila* resulted in a decrease in the number of DA neurons within the dorsomedial cluster (DMC) of 25-day-old PD model flies to 62.6% of that of age-matched control flies, while 1-day-old PD model and control flies showed no significant difference [137]. The reduction of dopamine level in the PD model fly brain was more prominent, such that at 10 days of age PD model flies showed a more than 50% reduction of brain dopamine level compared to control flies [137]. The number of DA neurons in the DMC of PD model flies treated with 5 *μ*g/mL of celastrol was significantly increased over that in the DMC of untreated control flies [137]. Moreover, treatment of flies with 5 *μ*g/mL of celastrol significantly increased the dopamine level [137].

The neuroprotective effect of celastrol was also demonstrated in the 1-methyl-4-phenyl-1,2,3,6-tetrahydropyridine (MPTP)-injected mouse model of PD [142]. Celastrol significantly attenuated the DA neuron loss in the substantia nigra pars compacta and the depletion of striatal dopamine induced by MPTP. More recently, a study has shown that pretreatment with celastrol enhanced cell viability and decreased cell apoptosis in rotenone-treated SH-SY5Y cells [143]. In this study, celastrol increased the LC3-II/LC3-I ratio, indicating that celastrol activated autophagic pathways. Moreover, inhibiting autophagy with 3-methyl adenine abolished the protective effects of celastrol, suggesting that celastrol protects SH-SY5Y cells from rotenone-induced injuries through induction of autophagy [143].

The effects of celastrol on locomotor activity and oxidative stress response were also investigated using the* Drosophila DJ-1*α** RNAi PD model. As observed in other* Drosophila* PD models [26–28, 81], the* DJ-1*α**RNAi PD model flies exhibited locomotor dysfunction as measured by a reduction of climbing activity [137]. After celastrol treatment for 20 days, the climbing activity of the* DJ-1*α** RNAi PD model flies was significantly improved [137]. Moreover, celastrol also significantly improved the survival of the* DJ-1*α** deletion mutant,* DJ-1α* ^Δ72^, under H_2_O_2_-induced oxidative stress [137]. Collectively, the studies in* Drosophila* and mammalian PD models suggest that celastrol can protect DA neurons against insults caused by various genetic or chemical factors and that* Drosophila* PD models are valuable for pharmacological studies.

### 3.4. Effect of Curcumin on* Drosophila *Models of AD and PD

Curcumin, a polyphenol extracted from the rhizome of the plant* Curcuma longa*, is widely used in Southeast Asia, China, and India for food and medicinal purposes [144]. Interestingly, an epidemiological study of Indian populations in which curcumin is consumed on a continual basis showed that the incidence of AD was 4.4-fold lower in these populations than in the USA [145], and numerous studies have established the neuroprotective effect of curcumin* in vivo* and* in vitro* [144]. In* Drosophila*, consumption of curcumin or its active metabolite tetrahydrocurcumin extends lifespan [146, 147] as in mice [148] and suppresses neurological phenotypes produced in the flies by chronic exposure to acrylamide and by reducing neuronal damage [149].

Recently, the potency of curcumin in alleviating A*β* neurotoxicity was investigated in transgenic* Drosophila* models of AD [94]. Curcumin feeding significantly improved the lifespan and locomotor activity of wild type or E22G mutant A*β*42-expressing flies [94]. Interestingly, curcumin accelerated amyloid oligomer-to-fibril conversion in* Drosophila* brain, and consistent with this result,* in vitro* aggregation of A*β*42 was enhanced in the presence of curcumin, which suggests that curcumin exerts its neuroprotective activity against A*β*42 by aggregating the neurotoxic oligomers into amyloid fibrils [94].

In addition, several studies have shown that curcumin also has therapeutic effects against PD and has anti-inflammatory and antioxidant activity in various* in vitro* and* in vivo* models of PD [150–153]. Curcumin rescued rotenone-induced cell death in SH-SY5Y cells and inhibited the aggregation and oligomerization of *α*-syn in SH-SY5Y cells [150, 153]. Moreover, treatment with curcumin remarkably improved behavioral disorders and survival of DA neurons in the MPTP mouse model of PD [154, 155]. The neuroprotective effect of curcumin has also been evaluated in* Drosophila* PD models [152, 153]. Curcumin promoted the survival of rotenone-treated PD model flies by reducing the intracellular and mitochondrial ROS levels [153] and ameliorated PD-like phenotypes by reducing ROS levels and inactivating LRRK2, a PD-associated protein [152]. Curcumin rescued the loss of DA neurons and reduction of dopamine levels in the brain of the PD fly and significantly improved climbing ability, of which loss is one of the representative phenotypes of PD [152, 153]. Moreover, in**α*-syn*-expressing PD flies, increased brain oxidative stress and apoptosis and sleep-deprivation-induced long-term learning deficits were successfully prevented when the flies were treated with curcumin throughout their lives [156, 157]. These results suggest that curcumin could be used for treating or preventing various types of PD.

Taken together, these studies show that the beneficial effect of curcumin against both AD and PD has been well established in several model systems. Among them,* Drosophila* has played an important role in this achievement.

## 4. Conclusion

Because it is an excellent genetic model system,* Drosophila* has been widely adopted for studies on most biological processes, including the pathology of human diseases. Based on the availability of various powerful tools of both genetics and molecular biology, the fly system should be a useful alternative model for pharmacological studies on the effects of traditional medicines on the pathology of neurodegenerative diseases. Yet surprisingly, only a limited number of studies have been performed to date in this field using* Drosophila *models. This may be due to relatively low physiological coherence between Drosophila and human compared to coherence between mouse or human cell models and human. In addition, the difference in diet between* Drosophila* and mammals also complicates the use of* Drosophila* in the study of traditional medicines, which are mostly dependent on natural nutrients. Despite these obstacles, a growing body of evidence supports the notion that a large portion of the pathophysiology of neurodegenerative diseases is well conserved in* Drosophila*. Moreover,* Drosophila* is a simple* in vivo* metazoan model, which can be used for evaluating the efficacy of a drug at various levels: whether it enters an animal body, targets neurons, or exerts its protective effect in not only cells but also in tissues. Therefore,* Drosophila* has been successfully used for identifying new drug candidates for the neurodegenerative diseases and for evaluating the efficacy and safety of these candidates. These successes in drug development highlight the enormous potential of* Drosophila* as a tool for the pharmacological study of traditional medicines.

## Abbreviations

A*β*: Amyloid *β*-peptide
AD: Alzheimer's disease
DA: Dopamine
PD: Parkinson's disease
SHXW: SuHeXiang Wang
UAS: Upstream activation sequence.
